# Effects of Lee Silverman Voice Treatment BIG and conventional physiotherapy on non-motor and motor symptoms in Parkinson’s disease: a randomized controlled study comparing three exercise models

**DOI:** 10.1177/1756286420986744

**Published:** 2021-02-18

**Authors:** Fabian Schaible, Franziska Maier, Timo Marcel Buchwitz, Frank Schwartz, Marius Hoock, Eckhard Schönau, Miriam Libuda, Anke Hordt, Thilo van Eimeren, Lars Timmermann, Carsten Eggers

**Affiliations:** Department of Neurology, University Hospital of Cologne, Cologne, Germany; Department of Psychiatry, University Hospital of Cologne, Medical Faculty, Cologne, Germany; Department of Neurology, University Hospital of Marburg, Marburg, Germany; Department of Neurology, University Hospital of Cologne, Cologne, Germany; Department of Neurology, University Hospital of Cologne, Cologne, Germany; Pediatric Endocrinology, University Hospital of Cologne, Cologne, Germany University of Cologne, Medical Faculty and University Hospital, Center of Prevention and Rehabilitation, UniReha, Germany; University of Cologne, Medical Faculty and University Hospital, Center of Prevention and Rehabilitation, UniReha, Germany; University of Cologne, Medical Faculty and University Hospital, Center of Prevention and Rehabilitation, UniReha, Germany; Department of Nuclear Medicine, University Hospital of Cologne, Cologne, Germany; Department of Neurology, University Hospital of Marburg, Marburg, Germany; Department of Neurology, University Hospital Marburg, Baldingerstr. 1, Marburg 35033, Germany Marburg Center of Mind, Brain and Behavior, Marburg, Germany

**Keywords:** LSVT BIG, non-motor symptoms, motor symptoms, Parkinson’s disease, physiotherapy

## Abstract

**Background:**

Parkinson’s disease (PD) patients experience disabling motor dysfunctions as well as non-motor symptoms (NMSs) that can highly impact their perceived quality of life. Besides pharmacological treatment options, active intervention programs have set some attention in managing these symptoms. However, previous studies mainly assessed the effectiveness of active intervention programs on functional mobility and motor symptoms.

**Objective:**

This study aimed to investigate the effect of Lee Silverman Voice Treatment (LSVT) BIG, an intensified and personalized physiotherapy (INTENSIVE), and a conventional physiotherapy (NORMAL) on NMSs in PD.

**Method:**

Forty-four patients with mild to moderate PD were randomly assigned to one of the three treatment groups. LSVT BIG and INTENSIVE were delivered one-on-one in 16 1-hour sessions within 4 weeks (4×/week). Patients assigned to NORMAL received 16 individual 1-hour sessions within 8 weeks (2×/week). The primary outcome measure was the difference in change from baseline in the non-motor symptom assessment scale for Parkinson’s disease (NMSS) between treatment groups to follow up at week 8. Patients were blinded for the NMSS being the primary outcome, but not the different treatment groups.

**Results:**

ANCOVA (Analysis of Covariance) showed reduced NMSS scores for all groups, with INTENSIVE being superior to NORMAL (*p* = 0.033). For secondary outcome measures (stride length, gait velocity and chair rising test) LSVT BIG and INTENSIVE were both superior to NORMAL.

**Conclusions:**

The study provides evidence that all three exercise programs are effective techniques to improve NMSs as well as motor function in PD.

**DRKS registration number:**

DRKS00008732

## Introduction

Parkinson’s disease (PD) is a frequent neurodegenerative disorder, reducing patients’ health-related quality of life in the course of the disease.^1^ Although traditionally conceptualized as a motor disorder, PD is also related to a variety of non-motor symptoms (NMSs). Most commonly described primary NMSs of PD are autonomic dysfunction, cognitive deficits, mood disturbances, sensory dysfunction, pain, and sleep disorders.^2,3^ These NMSs negatively affect patients’ perceived quality of life and can be even more disabling than motor dysfunctions.^4,5^ Pharmacological or even neurosurgical treatment often provides symptomatic relief but deficits sometimes cannot be controlled satisfactorily or side effects of treatment appear and worsen symptoms.^6^ Some NMSs such as sensory dysfunction, excessive sweating and anxiety completely lack an evidence-based pharmacological treatment option.^7^ Therefore, new non-pharmacological treatments are necessary to treat patients.

As such, therapeutic options like active intervention programs; for example, dance and music interventions, boxing sessions, Nordic walking or resistance training have already shown promising results in controlling and improving motor dysfunctions.^8–12^ Especially the amplitude-specific Lee Silverman Voice Treatment BIG (LSVT BIG) therapy has shown positive effects for patients with PD, being more effective than general exercise, Nordic walking and a shortened LSVT BIG protocol in terms of the UPDRS (United Parkinson’s Disease Rating Scale) motor score.^13^ Furthermore, non-amplitude-specific (conventional) physiotherapy has been advocated for patients with PD. There is a large body of evidence showing the benefits of physiotherapy in terms of improvements in walking, balance, muscle strength or reducing falls.^14–16^ Conventional physiotherapy thereby aims to preserve, enhance or restore movements and physical functions impaired or threatened by disease, injury and disability. Training techniques encompass active exercise modalities such as aerobic endurance and muscle strength training, cueing techniques and cognitive movement strategies to improve limitations in physical capacity, gait, balance, posture and transfer.^17^ The main focus of the majority of all these previous studies was placed on motor difficulties such as gait disturbances and balance issues. Recently published studies support a potential benefit of exercises for alleviating NMSs, including mood, sleep and cognition.^18^ For example, a 12-week progressive resistance training shows positive effects on cardiovascular autonomic dysfunction in PD by reduced systolic blood pressure during orthostatic stress and reduced heart rate variability.^19^ Studies about multidisciplinary intensive rehabilitation treatment, Yoga and Qigong indicate that physical activity can also improve sleep quality and reduce depression levels in PD.^20–22^ Further clinical trials brought evidence that various exercise modalities including aerobic and resistance training have beneficial effects on cognitive domains such as executive functions and memory.^23^ Although these results provide evidence that exercise promotes improvements in both motor and NMSs it is still unclear how frequently exercise should be performed for maximal benefit and which form of exercise is most effective in improving motor and NMSs.

With LSVT BIG showing promising results in improving motor features and conventional physiotherapy being most frequently applied in PD patients, this 8-week randomized controlled study aimed to compare three exercise models (specific protocol *versus* individualized protocol *versus* standard care) and their effects on NMSs that have not yet been extensively studied. By comparing LSVT BIG to an equally intensive physiotherapeutic training program we furthermore examined in how far the specific protocol of LSVT BIG or the high-intensity individualized training program itself is crucial for improved outcomes. The rationale to choose an 8-week observation period for all three groups was due to the fact that the total amount of received therapies should be comparable. This was oriented on the frequency of NORMAL physiotherapy which reflects the current (German) standard of care.

## Method

### Patients

Patients were screened from local support groups, general neurologists and the University Hospital of Cologne (UHoC) between July 2015 and May 2017. Participants were required to fulfill diagnostic criteria according to the latest Movement Disorder Society clinical diagnostic criteria for idiopathic PD.^24^ Inclusion criteria were Hoehn & Yahr stages I–III, age between 35 and 80 years, no walking aids, stable medication 4 weeks prior to and during the study. Exclusion criteria were dementia (PANDA (Parkinson’s Neuropsychometric Dementia Assessment) < 14), depression (BDI (Beck Depression Inventory) > 28), antidepressive or antipsychotic medication, participation in a LSVT BIG therapy in the past year, disabling bradykinesia to ensure patients are able to participate in the intensive physiotherapy (based on clinical impression and in accordance to UPDRS part III Item 14) and prior history of cardiovascular, neurological or musculoskeletal disorders known to interfere with testing PD features. Patients were not blinded in regards to the different study groups as all patients were educated about the study design while checking for eligibility. However, they were not specifically informed about the primary outcome of the study or a potential superiority of one of the groups. Patients who met inclusion criteria were randomly assigned in a 1:1:1 ratio to LSVT BIG, INTENSIVE or NORMAL physiotherapy. Research staff without clinical or research involvement in the study performed randomization using an online randomization software (www.sealedenvelope.com) which generated a random numbers table with a randomized allocation sequence.

The study was approved by the ethics committee of the Medical Faculty of the University of Cologne (registration number EK-15-200). In addition, the trial was registered in the German registry for clinical trials (DRKS number DRKS00008732). All participants gave written informed consent prior to data collection. The study was conducted according to the Declaration of Helsinki.

### Interventions

Patients assigned to LSVT BIG received 16 individual 1-hour-sessions (four times a week for 4 weeks) at the UHoC. The same physiotherapist (A.H.) who was a qualified LSVT BIG therapist with 6 years LSVT BIG experience delivered all LSVT BIG sessions. The training structure of LSVT BIG has previously been described.^25,26^ Briefly, half of the treatment sessions consist of standardized multidirectional whole-body movements performed with maximal amplitude of reaching and stepping. The second half is designed to address individual deficits in movement that occur in activities of daily living. Exercises progress in difficulty by increasing range of movement, duration and complexity. The physiotherapist is instructed to provide motivation to patients during every repetition of a movement. Feedback about the movement amplitude is given to each patient individually and patients are instructed to include larger movements in real-life situations in order to achieve practice in everyday movements.

Patients assigned to intensive physiotherapy received 16 individual 1-hour-sessions (four times a week for 4 weeks) at the UHoC. Three PD-experienced physiotherapists (M.L. 12 years’ experience, A.B. 8 years’ experience, A.H. 13 years’ experience) delivered the sessions and constantly encouraged patients to work with at least 60% to 80% of their maximal effort. The Borg scale was used to rate the perceived exertion.^27^ Training was conducted according to the European Guideline for Parkinson’s Physiotherapy with special focus on assessment of individual deficits, gait, falls, freezing of gait and dexterity and the according practice considerations.^28^ The training program was individually elaborated for each patient and encompassed a set of exercises which varied among the participants according to their own needs. These included, for example, complex motor sequences, stretching items to increase mobility, dual tasks, core stability or mental imagery. To support the ability to exercise at home several worksheets explaining the afore-mentioned exercises were prepared and given to the patients. As these worksheets also were regularly used during the sessions of intensive physiotherapy, they are accessible in Supplemental Appendix 1. Due to the individual training sets no overall number of exercises or repetitions could be calculated. Intensity gradually progressed over the sessions by increasing the number of repetitions, weights, difficulty of tasks and pace with increases in the Borg scale (medium 6–10 → high intensity 17 points), increases in the heart rate or numbers of repetitions.

Patients assigned to normal physiotherapy received 16 1-hour-sessions (two times a week for 8 weeks). Patients were allowed to undergo training in an office-based physiotherapy practice of their choice outside of the premises of the UHoC. No special exercises were prescribed nor the number of repetitions or resistance levels observed. This group was aimed to reflect the current standard physiotherapeutic treatment of PD in office-based practice.

LSVT BIG and INTENSIVE physiotherapy groups were held in individual one-on-one training sessions at the UHoC on different days. Patients assigned to LSVT BIG or INTENSIVE physiotherapy were encouraged to practice steadily at home for at least 4 and not more than 6 hours per week. All subjects were instructed not to participate in additional activities throughout the 8-week intervention period. At the end of the training periods patients had to confirm that they did not do additional activities. Training programs were delivered individually with all groups receiving in total the same amount of the therapist’s time. The frequency of therapy differed between LSVT BIG/INTENSIVE (4×/week for 4 weeks) and NORMAL (2×/week for 8 weeks). As stated earlier patients were not blinded to the therapy groups; however, they did not receive any information about the potential superiority of one of the groups.

### Outcomes

**Figure 1.**
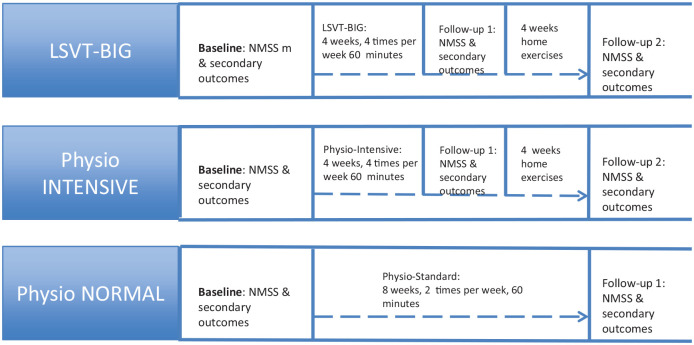
Overview of tests and times per group.

All outcome parameters were assessed in the week before training started (pre-test/baseline) and after 8 weeks (follow-up; see Figure 1). An additional follow-up for LSVT BIG and intensive physiotherapy was done at the end of the 4-week training period. All patients were tested on their regular medication in the “on” medication state.

The primary outcome parameter was the difference in change of NMSs between treatment groups using the non-motor symptom assessment scale for Parkinson’s disease (NMSS) between baseline and 8 weeks follow-up. The NMSS is known to have a high test–retest correlation (intraclass correlation coefficient (ICC) >0.9) and is a tool which can be used for serial administration.^29^ The NMSS is administered and coded by the examiner and contains 30 items which are grouped into nine dimensions: cardiovascular (two items), sleep/fatigue (four items), mood/cognition (six items), perceptual problems/hallucinations (three items), attention/memory (three items), gastrointestinal (three items), urinary (three items), sexual function (two items), and miscellany (four items). The score for each item is based on a multiple of severity (from 0 to 3) and frequency scores (from 1 to 4).^30^ The range of total scores is 0 to 360. In the present study the examiner (F.S.) was not blinded to the NMSS being the primary outcome.

Secondary outcome variables included changes from baseline to 8 weeks follow-up in motor severity according to the UPDRS part III motor score, chair rising test and a force-measuring gangway. Patients were videotaped while performing all UPDRS part III items except rigidity. An independent experienced rater (M.H.) evaluated the videos blinded for group allocation and time of examination. The rating of rigidity was done by the person performing the physical examination (F.S.). Gait parameters (walking speed and step length) were measured using a 6-meter Leonardo Mechanograph Gangway system (Novotec Medical GmbH, Pforzheim, Germany). This system has a high test–retest reliability with an ICC > 0.9.^31^ The first and last step were excluded from the analysis as they are usually shorter than the average step length. The results of three consecutive walks with shoes were averaged after normalization for the number of steps. A minimum of four steps and a maximum of 12 steps per walk for each patient depending on stride length was recorded. The chair rising test was performed on a Leonardo Mechanograph GRFP system (Novotec Medical GmbH, Pforzheim, Germany; ICC > 0.9).^32^ The main parameter measured by the chair rising test was the time required for five chair rises with shoes. The results of three measurements were then averaged. Qualitative assessment of NMSs as a secondary outcome parameter was conducted using the revised final version of the PD NMS questionnaire.^33^ Further secondary outcome variables were quality of life (Parkinson’s Disease Questionnaire (PDQ-39)) and psychometric and cognitive functions (Beck Depression Inventory (BDI-2), Apathy Evaluation Scale (AES), Parkinson Neuropsychometric Dementia Assessment (PANDA), Mini Mental Status Test (MMST)). Daily medication was converted to the levodopa equivalence dose (LED) according to published conversion rates.^34^ As safinamide has no published conversion rates, the equivalence calculation for amantadine was used.

### Statistical analysis

The initial power calculation suggested a sample size of 60 patients (20 LSVT BIG, 20 INTENSIVE and 20 NORMAL) to be enrolled to test for a 7-point difference on the primary outcome (NMSS) with a standard deviation of 10 points, a level of significance of 0.05 and with a power of 80%. The target number included an estimated drop-out rate of 10%. The study was not powered to assess secondary outcomes.

Univariate ANOVA (Analysis of Variance) was used to detect significant differences in baseline characteristics (see Table 1). The equality of the variances in each group was verified with the Levene test. The chi-square test was used to check for gender differences. Differences in change from baseline to follow-up at week 8 between treatment groups were then assessed using analysis of covariance (ANCOVA) with the baseline values as a covariate. If ANCOVA revealed significant differences between groups pairwise post-hoc comparisons were performed. Data of the primary outcome analyses were corrected by the multiple comparison Bonferroni test. Values are reported as means with standard deviation (SD). In addition, differences in change from baseline to follow-up at week 4 (interim analysis) between LSVT BIG and INTENSIVE were similarly assessed using ANCOVA. If variables were not normally distributed, equivalent non-parametric tests were applied instead. To investigate further within-person changes of non-motor symptoms, Wilcoxon paired-rank tests were applied to NMSS subscore scales and secondary non-motor symptom assessments (AES, BDI, MMST, PANDA).

**Table 1.** Subject characteristics (values are means (SD), calculation with univariate ANOVA).

	LSVT-BIG (*n* = 14)	Intensive (*n* = 15)	Normal (*n* = 12)	*p*-value
	Mean and SD	Mean and SD	Mean and SD	
Baseline characteristics:				
Age in years	63.29 (8.48)	66.20 (8.65)	65.50 (8.21)	0.635
Disease duration since diagnosis in years	5.36 (2.59)	5.27 (3.41)	5.42 (4.21)	0.993
LED	405 (259)	418 (302)	497 (278)	0.670
Hoehn & Yahr stage	2.00 (0.71)	1.73 (0.68)	2.12 (0.43)	0.260
**Primary outcome parameters:**				
NMSs assessment scale for Parkinson’s disease total score	39.93 (18.35)	36.07 (23.65)	50.17 (16.03)	0.189
**Secondary outcome parameters:**				
PD NMSs questionnaire total score	8.43 (4.03)	6.87 (4.70)	8.42 (3.45)	0.517
PDQ-39 total score	19.07 (10.99)	15.00 (7.72)	22.85 (9.34)	0.111
UPDRS part III total score	26.79 (9.82)	22.60 (7.15)	28.67 (8.38)	0.172
PANDA total score	26.93 (1.54)	25.47 (4.19)	24.92 (2.75)	0.231
MMST total score	29.07 (0.92)	28.87 (1.19)	28.58 (1.73)	0.634
BDI-2 total score	8.21 (5.38)	4.87 (3.56)	9.67 (6.80)	0.062
AES total score	28.36 (6.22)	25.00 (6.69)	28.00 (7.52)	0.356
Chair rising test with shoes (av. total time in seconds)	12.88 (4.01)	10.68 (2.18)	12.52 (2.96)	0.144
Step length (m)	0.640 (0.103)	0.667 (0.093)	0.672 (0.879)	0.650
Walking velocity (m/sec.)	1.444 (0.232)	1.459 (0.231)	1.433 (0.186)	0.956

AES, Apathy Evaluation Scale (range 18–72; a lower score indicating greater apathy); ANOVA, analysis of variance; BDI, Beck Depression Inventory (range 0–63; a higher score indicating more signs of depression); Chair Rising Test, results for five repetitions; LED, Levodopa equivalent dose; MMST, Mini Mental Status Examination (range 0–30; a lower score indicating more cognitive impairment); NMSs, non-motor symptoms; PANDA, Parkinson Neuropsychometric Dementia Assessment (range 0–30; a lower score indicating more cognitive impairment); PD NMSs, Parkinson’s disease non-motor symptoms (range 0–360; a higher score indicating higher severity of non-motor symptoms); PDQ-39, Parkinson’s Disease Quality of Life Questionnaire (range 0–100; a higher score indicating more reduction in quality of life); SD, standard deviation; UPDRS, United Parkinson’s Disease Rating Scale (0–108; a higher score indicating more motor impairment).

The alpha level was set at 0.05 and primary and secondary efficacy analyses were conducted on an intention-to-treat (ITT) basis using IBM SPSS statistics version 25 software. The ITT population consisted of all patients who received the full amount of 16 individual 1-hour training sessions and who took part in at least one follow-up visit applying the last observation carried forward analysis in the case of missing values.

## Results

### Patient characteristics

**Figure 2.**
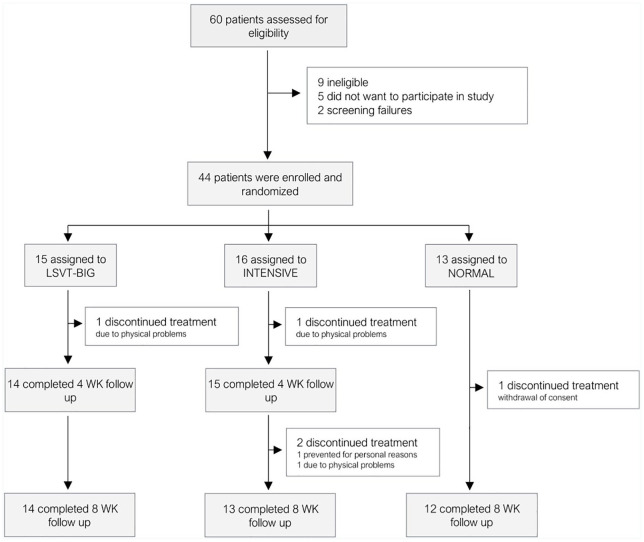
Consort flow diagram.

A total of 60 patients was screened for eligibility. Forty-four fulfilled the inclusion criteria and agreed to participate in the study. These were randomly assigned for one treatment group. Thirty-nine subjects completed the study and were available for follow-up at week 8 (drop-out rate 11%). Three patients dropped out of the INTENSIVE group (one patient discontinued treatment due to hip problems in week 3, two patients were not able to attend the second follow-up because of pneumonia and personal circumstances). One patient in LSVT BIG discontinued treatment in week 2 because of pneumonia and one patient in NORMAL withdrew written consent after 2 weeks. No adverse events were reported. In total, 41 patients were included in the ITT analysis: LSVT-BIG (*n* = 14), INTENSIVE (*n* = 15) and NORMAL (*n* = 12). The 16 screened patients who did not participate did not fulfill the inclusion criteria (*n* = 11) or were not interested in or did not see the need of a physiotherapeutic training program within a study (*n* = 5), see Figure 2.

The sample consisted of 23 men (56.1%) and 18 women (43.9%). These were distributed among the three different treatment groups as follows: LSVT-BIG (seven men/seven women), INTENSIVE (six men/nine women), NORMAL (10 men/two women). There was no significant difference in gender between the treatment groups [χ²(2) = 5.4, *p* = 0.067, *V* = 0.67]. Univariate ANOVA showed no significant differences between groups for disease duration, demographic aspects, LED, Hoehn & Yahr stages and baseline outcome parameters. Testing was conducted in the on-medication state. There were no adjustments of anti-Parkinsonian medication between baseline and follow-up at week 8 in any patient. The baseline characteristics of patients are listed in Table 1.

### Primary outcome

Results for the primary outcome parameter NMSS are shown in Table 2. ANCOVA showed a significant effect on the mean change of the NMSS total score between baseline and 8 weeks of INTENSIVE in comparison to NORMAL (−12.40, 95% CI (Confidence Interval) −24.00 to −0.79, *p* = 0.033). There was no significant effect on the mean change of the NMSS total score between baseline and 8 weeks of LSVT BIG in comparison to NORMAL (−4.06, 95% CI −15.62 to 7.49, *p* = 1.000) or INTENSIVE (8.33, 95% CI −2.38 to 19.05, *p* = 0.176). Numerically, there was a greater improvement of the NMSS total score in the INTENSIVE group (−19.04, 95% CI −25.12 to −12.95) than in the LSVT BIG group (−10.70, 95% CI −16.91 to 4.49). However, this change did not reach statistical significance. The Kruskal–Wallis test revealed no significant differences in mean change of NMSS subscores between groups. Wilcoxon paired-rank tests revealed significant within-group changes of NMSS subscores cardiovascular (−1.40, 95% CI −2.82 to 0.02, *p* = 0.043), sleep/fatigue (−4.53, 95% CI −8.02 to −1.04, *p* = 0.005), mood/cognition (−2.20, 95% CI −5.09 to 0.69, *p* = 0.042) and miscellany (−4.00, 95% CI −7.87 to −0.13, *p* = 0.036) for INTENSIVE after 8 weeks. The respective test results are listed in Table 3 (Supplemental Appendix 1). Interim analysis of the primary outcome revealed there was no significant difference of the mean change between baseline and 4 weeks for LSVT BIG compared to INTENSIVE (5.84, 95% CI −2.80 to 14.49, *p* = 0.177). Within-group changes of NMSS subscale sleep/fatigue were significant for INTENSIVE (−3.93, 95% CI −7.97 to 0.10, *p* = 0.004). INTENSIVE (−5.00, 95% CI −9.18 to −0.82, *p* = 0.025) and LSVT BIG (−3.86, 95% CI −7.18 to 0.53, *p* = 0.006) both reported significant within-group changes for NMSS subscore miscellany after 4 weeks.

**Table 2.** Overview of normally distributed outcome measures (ANCOVA with baseline values as covariate for mean changes from baseline to follow-up at 8 weeks).

	In groups					Between groups			
	Group	Mean change	95% CI			Mean change	95% CI		*p* value
			Lower	Upper			Lower	Upper	
**Primary outcome parameters:**									
NMSs assessment scale for Parkinson’s disease	BIG (*n* = 14)	−10.70	−16.91	−4.49	BIG *versus* INTENSIVE	8.33	−2.38	19.05	0.176
	INTENSIVE (*n* = 15)	−19.04	−25.12	−12.95	INTENSIVE *versus* NORMAL	−12.40	−24.00	−0.79	**0.033**
	NORMAL (*n* = 12)	−6.64	−13.54	0.26	NORMAL *versus* BIG	4.06	−7.49	15.62	1.000
**Secondary outcome parameters:**									
PD NMSs questionnaire	BIG	−2.39	−3.72	−1.06	BIG *versus* INTENSIVE	1.23	−0.64	3.10	0.190
	INTENSIVE	−3.62	−4.91	−2.33	INTENSIVE *versus* NORMAL	−2.35	−4.30	−0.41	**0.019**
	NORMAL	−1.27	−2.70	0.17	NORMAL *versus* BIG	1.12	−0.83	3.07	0.250
PDQ-39	BIG	−4.08	−7.47	−0.69	BIG *versus* INTENSIVE	2.77	−2.02	7.56	0.249
	INTENSIVE	−6.85	−10.22	−3.48	INTENSIVE *versus* NORMAL	−1.54	−6.74	3.66	0.552
	NORMAL	−5.31	−9.08	−1.54	NORMAL *versus* BIG	−1.23	−6.28	3.82	0.625
UPDRS part III	BIG	−4.58	−6.61	−2.55	BIG *versus* INTENSIVE	1.12	−1.76	4.00	0.434
	INTENSIVE	−5.70	−7.71	−3.69	INTENSIVE *versus* NORMAL	−3.60	−6.66	−0.53	**0.023**
	NORMAL	−2.11	−4.34	0.12	NORMAL *versus* BIG	2.47	−0.52	5.46	0.103
Chair rising test with shoes (av. Total time in seconds)	BIG	−2.74	−3.41	−2.06	BIG *versus* INTENSIVE	−0.55	−1.49	0.41	0.253
	INTENSIVE	−2.19	−2.83	−1.56	INTENSIVE *versus* NORMAL	−1.64	−2.59	−0.68	**0.001**
	NORMAL	−0.55	−1.25	0.14	NORMAL *versus* BIG	2.18	1.22	3.14	**<0.001**
Gangway with shoes (av. length per step (m)	BIG	0.068	0.045	0.092	BIG *versus* INTENSIVE	0.002	−0.030	0.034	0.901
	INTENSIVE	0.066	0.044	0.089	INTENSIVE *versus* NORMAL	0.047	0.014	0.081	**0.007**
	NORMAL	0.019	−0.006	0.044	NORMAL *versus* BIG	−0.049	−0.084	−0.015	**0.006**
Gangway with shoes (av. path length (m)/seconds)	BIG	0.185	0.107	0.262	BIG *versus* INTENSIVE	−0.044	−0.152	0.064	0.416
	INTENSIVE	0.228	0.153	0.303	INTENSIVE *versus* NORMAL	0.159	0.046	0.271	**0.007**
	NORMAL	0.070	−0.014	0.154	NORMAL *versus* BIG	−0.115	−0.229	−0.001	**0.049**

Significant *p*-values are highlighted in bold.

Between-groups *p*-values of the primary outcome have been corrected for multiple comparison using Bonferroni correction.

ANCOVA, analysis of covariance; CI, confidence interval; NMSs, non-motor symptoms; PD NMSs, Parkinson’s disease non-motor symptoms (range 0–360; a higher score indicating higher severity of non-motor symptoms); PDQ-39, Parkinson’s Disease Quality of Life Questionnaire (range 0–100; a higher score indicating more reduction in quality of life); UPDRS, United Parkinson’s Disease Rating Scale (0–108; a higher score indicating more motor impairment).

**Table 3.** Outcome measures for secondary outcomes and NMSs subscales in and between groups (Kruskal–Wallis test for mean changes between groups and Wilcoxon signed-rank test for mean changes within groups from baseline to follow-up at 8 weeks).

	Group	In groups				Between groups	
		Mean change	95% CI		*p*-value	Mean rank	*p*-value
			Lower	Upper			
**Secondary outcomes:**							
AES	BIG (*n* = 14)	−2.43	−6.11	1.26	0.151	18.86	0.704
	INTENSIVE (*n* = 15)	−1.80	−4.63	1.03	0.164	21.87	
	NORMAL (*n* = 12)	−1.17	−3.93	1.59	0.442	22.42	
BDI							0.336
	BIG	−2.21	−3.56	−0.87	**0.004**	19.61	
	INTENSIVE	−1.20	−2.56	0.16	0.074	24.53	
	NORMAL	−2.17	−4.23	−0.11	**0.045**	18.21	
MMST							0.388
	BIG	0.21	−0.61	1.04	0.464	19.54	
	INTENSIVE	0.87	0.28	1.45	**0.010**	24.27	
	NORMAL	0.42	−1.00	1.84	0.716	18.63	
PANDA							0.250
	BIG	0.36	−1.24	1.95	0.380	17.54	
	INTENSIVE	2.67	0.53	4.81	**0.003**	24.77	
	NORMAL	1.33	−1.06	3.73	0.234	20.33	
**NMSs subscales:**							
Cardiovascular							0.616
	BIG (*n* = 14)	−0.71	−2.08	0.65	0.340	23.29	
	INTENSIVE (*n* = 15)	−1.40	−2.82	0.02	**0.043**	19.87	
	NORMAL (*n* = 12)	−1.25	−2.69	0.19	0.074	19.75	
Sleep/fatigue							0.385
	BIG	−1.36	−2.90	0.19	0.079	24.43	
	INTENSIVE	−4.53	−8.02	−1.04	**0.005**	18.43	
	NORMAL	−2.67	−6.65	1.32	0.126	20.21	
Mood/cognition							0.927
	BIG	−1.29	−3.84	1.27	0.260	21.04	
	INTENSIVE	−2.20	−5.09	0.69	**0.042**	20.20	
	NORMAL	−1.75	−5.87	2.37	0.532	21.96	
Perceptual problems/hallucinations							0.515
	BIG	0.29	−029	0.86	0.285	21.43	
	INTENSIVE	−0.73	−2.40	0.93	0.416	18.90	
	NORMAL	0.42	−0.68	1.52	0.357	23.13	
Attention/memory							0.709
	BIG	−1.50	−3.83	0.83	0.098	19.21	
	INTENSIVE	−1.13	−2.50	0.24	0.112	21.03	
	NORMAL	−0.50	−2.90	1.90	0.673	23.04	
Gastrointestinal							0.262
	BIG	−1.50	−3.14	0.14	0.058	17.21	
	INTENSIVE	0.20	−0.53	0.93	0.480	23.73	
	NORMAL	−0.42	−2.79	1.95	0.725	22.00	
Urinary							0.866
	BIG	−0.64	−4.40	3.12	0.235	20.57	
	INTENSIVE	−2.13	−5.78	1.52	0.419	22.27	
	NORMAL	−2.33	−6.02	1.35	0.229	19.92	
Sexual function							0.879
	BIG	−0.43	−1.58	0.72	0.593	21.64	
	INTENSIVE	−1.07	−2.88	0.75	0.285	21.27	
	NORMAL	−0.83	−2.34	0.67	0.273	19.92	
Miscellany							0.593
	BIG	−3.00	−7.57	1.57	0.207	21.29	
	INTENSIVE	−4.00	−7.87	−0.13	**0.036**	18.77	
	NORMAL	−0.75	−4.49	2.99	0.475	23.46	

Significant *p*-values are highlighted in bold.

Between-groups *p*-values of the primary outcome have been corrected for multiple comparison using Bonferroni correction.

AES, Apathy Evaluation Scale (range 18–72; a lower score indicating greater apathy); BDI, Beck Depression Inventory (range 0–63; a higher score indicating more signs of depression); CI, confidence interval; MMST, Mini Mental Status Examination (range 0–30; a lower score indicating more cognitive impairment); NMSs, non-motor symptoms; PANDA, Parkinson Neuropsychometric Dementia Assessment (range 0–30; a lower score indicating more cognitive impairment).

### Secondary outcomes

Results for the significant changes of the secondary outcome parameters are shown in Tables 2 and 3. ANCOVA revealed a significant effect on the mean change of the PD NMSs questionnaire total score between baseline and 8 weeks of INTENSIVE in comparison to NORMAL (−12.40, 95% CI −24.00 to −0.79, *p* = 0.019). There was no significant effect on the mean change of the PD NMSs questionnaire total score between baseline and 8 weeks of LSVT BIG in comparison to NORMAL or INTENSIVE. ANCOVA results of the UPDRS part III total score revealed a significant effect on the mean change between baseline and 8 weeks of INTENSIVE in comparison to NORMAL (−3.60, 95% CI −6.66 to −0.53, *p* = 0.023). No significant effects on the mean change of the UPDRS part III total score were reported for LSVT BIG in comparison to INTENSIVE or NORMAL. Gait parameters improved significantly between baseline and 8 weeks of LSVT BIG and INTENSIVE in comparison to NORMAL [average length per step: LSVT BIG *versus* NORMAL (0.049, 95% CI 0.015−0.084, *p* = 0.006) and INTENSIVE *versus* NORMAL (0.047, 95% CI 0.014−0.081, *p* = 0.007); average pathlength/seconds: LSVT BIG *versus* NORMAL (0.115, 95% CI 0.001−0.229, *p* = 0.049) and INTENSIVE *versus* NORMAL (0.159, 95% CI 0.046−0.271, *p* = 0.007)]. There was no significant effect on the mean change of gait parameters between baseline and 8 weeks of LSVT BIG in comparison to INTENSIVE. For the chair rising test, ANCOVA revealed significant effects on the mean change between baseline and 8 weeks of LSVT BIG (2.18, 95% CI −3.14 to −1.22, *p* < 0.001) and INTENSIVE (−1.64, 95% CI −2.59 to −0.68, *p* = 0.001) in comparison to NORMAL. There were no significant group differences for any of the other secondary outcomes (PDQ-39, BDI-2, AES, MMST, PANDA). However, within-group comparisons between baseline and 8 weeks revealed significant changes of BDI-2 scores for LSVT BIG (−2.21, 95% CI −3.56 to −0.87, *p* = 0.004) and NORMAL (−2.17, 95% CI −4.23 to −0.11, *p* = 0.045), but not INTENSIVE. Within-group changes of INTENSIVE were significant for PANDA (2.67, 95% CI 0.53−4.81, *p* = 0.003) and MMST scores (0.87, 95% CI 0.28−1.45, *p* = 0.010). Interim analyses of the secondary outcomes showed no significant differences of the mean changes between baseline and 4 weeks for LSVT BIG compared to INTENSIVE. The Wilcoxon paired-rank test revealed a significant change of AES scores for both INTENSIVE (−2.00, 95% CI −4.70 to 0.70, *p* = 0.044) and LSVT BIG (−3.57, 95% CI −5.91 to −1.23, *p* = 0.011). The BDI-2 score change was significant for LSVT-BIG (−3.64, 95% CI −6.38 to −0.90, *p* = 0.014) only. The within-group change of the PANDA total score was significant for INTENSIVE (1.67, 95% CI −0.58 to 3.92, *p* = 0.040) and LSVT-BIG (1.14, 95% CI 0.27−2.02, *p* = 0.012). The change of the MMST score was significant for INTENSIVE (0.73, 95% CI 0.12 to 1.34, *p* = 0.014), but not LSVT BIG. Test results are listed in Tables 4 and 5.

**Table 4.** Outcome measures of LSVT BIG and INTENSIVE (ANCOVA with baseline values as covariate for mean changes from baseline to follow-up at 4 weeks).

	Group	In groups				Between groups			
		Mean change	95% CI			Mean change	95% CI		*p*-value
			Lower	Upper			Lower	Upper	
**Primary outcome parameters:**									
NMSs assessment scale for Parkinson’s disease	BIG (*n* = 14)	−12.14	−20.86	−3.43	BIG *versus* INTENSIVE	5.844	−2.80	14.49	0.177
	INTENSIVE (*n* = 15)	−16.07	−24.64	−7.49					
**Secondary outcome parameters:**									
PD NMSs questionnaire	BIG	−2.43	−4.24	−0.62	BIG *versus* INTENSIVE	1.314	−0.58	3.21	0.166
	INTENSIVE	−3.07	−4.72	−1.41					
PDQ-39	BIG	−10.57	−17.06	−4.08	BIG *versus* INTENSIVE	1.311	−5.46	8.08	0.694
	INTENSIVE	−8.27	−13.37	−3.16					
UPDRS part III	BIG	−4.86	−6.94	−2.77	BIG *versus* INTENSIVE	−0.113	−3.06	2.83	0.938
	INTENSIVE	−4.47	−6.57	−2.36					
Chair rising test with shoes (av. total time in seconds)	BIG	−2.77	−4.06	−1.48	BIG *versus* INTENSIVE	−0.323	−1.18	0.53	0.441
	INTENSIVE	−1.55	−2.12	−0.97					
Gangway with shoes (av. length per step (m)	BIG	0.09	0.05	0.14	BIG *versus* INTENSIVE	0.012	−0.03	0.05	0.572
	INTENSIVE	0.07	0.05	0.09					
Gangway with shoes (av. path length (m)/seconds)	BIG	0.25	0.13	0.38	BIG *versus* INTENSIVE	0.046	−0.09	0.19	0.506
	INTENSIVE	0.20	0.11	0.29					

Significant *p*-values are highlighted in bold.

ANCOVA, analysis of covariance; Chair Rising Test, results for five repetitions; CI, confidence interval; LSVT, Lee Silverman Voice Treatment; NMSs, non-motor symptoms; PD NMSs, Parkinson’s disease non-motor symptoms (range 0–360; a higher score indicating higher severity of non-motor symptoms); PDQ-39, Parkinson’s Disease Quality of Life Questionnaire (range 0–100; a higher score indicating more reduction in quality of life); UPDRS, United Parkinson’s Disease Rating Scale (0–108; a higher score indicating more motor impairment).

**Table 5.** Outcome measures for secondary outcomes and NMSs subscales in and between groups (Mann–Whitney U-test for mean changes between groups and Wilcoxon signed-rank test for mean changes within groups from baseline to follow-up at 4 weeks).

	Group	In groups				Between groups	
		Mean change	95% CI		*p*-value	Mean rank	*p*-value
			Lower	Upper			
Secondary outcomes:							
AES	BIG (*n* = 14)	−3.57	−5.91	−1.23	**0.011**	13.25	0.290
	INTENSIVE (*n* = 15)	−2.00	−4.70	0.70	**0.044**	16.63	
BDI	BIG	−3.64	−6.38	−0.90	**0.014**	12.00	0.070
	INTENSIVE	−0.60	−2.02	0.82	0.258	17.80	
MMST	BIG	0.36	−0.48	1.19	0.399	14.11	0.591
	INTENSIVE	0.73	0.12	1.34	**0.014**	15.83	
PANDA	BIG	1.14	0.27	2.02	**0.012**	14.18	0.621
	INTENSIVE	1.67	−0.58	3.92	**0.040**	15.77	
**NMSs subscales:**							
Cardiovascular	BIG (*n* = 14)	0.14	−0.81	1.10	0.785	16.11	0.505
	INTENSIVE (*n* = 15)	−1.20	−2.84	0.44	0.136	13.97	
Sleep/fatigue	BIG	−1.29	−3.71	1.14	0.238	16.36	0.425
	INTENSIVE	−3.93	−7.97	0.10	**0.004**	13.73	
Mood/cognition	BIG	−2.29	−5.15	0.57	0.065	13.89	0.505
	INTENSIVE	−2.73	−6.75	1.28	0.061	16.03	
Perceptual problems/hallucinations	BIG	0.43	−0.67	1.53	0.655	16.93	0.252
	INTENSIVE	−1.13	−2.61	0.34	0.066	13.20	
Attention/memory	BIG	−1.93	−4.23	0.38	0.129	14.21	0.652
	INTENSIVE	−0.80	−2.30	0.70	0.259	15.73	
Gastrointestinal	BIG	−1.07	−3.18	1.03	0.306	13.79	0.477
	INTENSIVE	−0.20	−0.93	0.53	0.581	16.13	
Urinary	BIG	−2.14	−5.55	1.27	0.234	13.64	0.425
	INTENSIVE	−0.20	−1.77	1.37	0.918	16.27	
Sexual function	BIG	−0.21	−1.37	0.94	0.593	15.57	0.747
	INTENSIVE	−1.00	−2.40	0.40	0.144	14.47	
Miscellany	BIG	−3.86	−7.18	−0.53	**0.006**	15.71	0.683
	INTENSIVE	−5.00	−9.18	−0.82	**0.025**	14.33	

Significant *p*-values are highlighted in bold.

AES, Apathy Evaluation Scale (range 18–72; a lower score indicating greater apathy); BDI, Beck Depression Inventory (range 0–63; a higher score indicating more signs of depression); MMST, Mini Mental Status Examination (range 0–30; a lower score indicating more cognitive impairment); NMSs, non-motor symptoms; PANDA, Parkinson Neuropsychometric Dementia Assessment (range 0–30; a lower score indicating more cognitive impairment).

## Discussion

In the current prospective, randomized controlled study we examined the impact of LSVT BIG therapy on motor and NMSs in PD and compared LSVT BIG to an intensified conventional physiotherapy and a normal conventional physiotherapy program. Our findings clearly indicate that participation in all three exercise models has beneficial effects on both motor and non-motor dysfunctions.

The degree of change in NMSS varies among the different exercise models, with INTENSIVE improving the most (mean change of −19.04) followed by LSVT BIG (mean change of −10.70) and NORMAL (mean change of −6.64). Between-group comparisons revealed a significant difference between INTENSIVE and NORMAL, indicating the superiority of more intensive physiotherapy compared to standard care with regard to total NMSs reduction. Interestingly, LSVT BIG did not differ significantly from NORMAL or INTENSIVE. Thus, intensive physiotherapy is not better suited to ameliorate NMSs in PD than LSVT BIG. Also, LSVT BIG is not more effective than standard care.

The examination of NMSS subscore changes after 8 weeks did not reveal any significant differences between groups at all. However, several significant within-group changes were found exclusively for INTENSIVE, but not the other two exercise groups. These changes over time comprise lower scores for the dimensions cardiovascular, sleep/fatigue, mood/cognition and miscellaneous. The results of previous studies support the positive effect of physical activity on various non-motor symptoms in PD.^35^ Interestingly, in our study changes of the subscale mood/cognition, which mainly asks about feelings similar to depressive symptoms, for INTENSIVE are not supported by within-group results of BDI-2. Oddly, significant changes of depressive symptoms measured by BDI-2 were only found for LSVT BIG after 4 weeks and LSVT BIG and NORMAL, but not for INTENSIVE, after 8 weeks. This could be due to both tests having a different scope and slightly different aims. Similarly, significant within-group improvements of cognition (measured by MMST and PANDA) were exclusively present for INTENSIVE. The fact that the NMSS subscale attention/memory is a self-reported measurement which focuses on very specific aspects of cognitive functioning, might explain why positive changes of general cognition are not reflected. While the existing heterogeneity of within-group results might hinder clear interpretation of non-motor symptom changes, between-group changes of NMSS subscales and other secondary outcome assessments are congruent. Thus, it is difficult to conclude whether intensive physiotherapy, compared to LSVT BIG and standard care, is superior in ameliorating these specific NMSs in PD, which might also be impactful with regard to quality of life.^36^

Pain is a common non-motor symptom in PD^37^ and there is a potential role for exercise in pain management programs. However, only one item in the NMSS subscale miscellaneous enquires about pain. It is unclear if the afore-mentioned within-group change of the dimension miscellaneous is related to a change of pain levels or another item on the subscale. Because of the complex nature of pain, it should be considered to be the main objective evaluated in future trials.

Up to now only one recently published study on Nordic walking has used the NMSS as an assessment tool to evaluate the effects of an exercise program on NMSs in PD.^10^ The results revealed a significant improvement of the overall NMSS score (mean change of −23.2) for 12 weeks of Nordic walking training in comparison to a control group. Although Nordic walking and the INTENSIVE group seem to cause a similar range of improvements in the NMSS score, results have to be interpreted with caution as the Nordic walking cohort was considerably more affected at baseline by NMSs [baseline NMSS score of 96 (SD 34.2) compared to 36.07 (SD 23.65) in our INTENSIVE group]. Further previously published studies addressing the effects of exercise on general NMSs used the UPDRS part I or MDS-UPDRS part I questionnaire; in this sense the results are not likewise comparable. Twelve months of 1-hour Tango sessions twice per week and a 24-week treadmill training showed improved MDS-UPDRS part I scores.^38,39^ A positive effect on NMSs was also displayed for 12 weeks of physiotherapy while a 3-year active theatre training improved the UPDRS part I score significantly in comparison with a control group receiving standard physiotherapy.^40,41^ Addressing the effects of exercise on NMSs, these preliminary data suggest positive effects on global NMSs for various forms of exercise. Our results support these findings and display the first randomized controlled trial focusing on NMSs as a primary outcome measure.

Secondary outcome data in terms of the assessment of the UPDRS part III motor score showed that INTENSIVE training was superior to NORMAL. A degree of change (mean −5.70) is considered as clinically relevant.^42^ With a mean change of −4.58 also the LSVT BIG UPDRS part III motor score improved clinically meaningfully but not significantly in comparison to NORMAL.

A further assessment of gait parameters and chair rising test performance observed superior results for both LSVT BIG and INTENSIVE in comparison to NORMAL, underlining potential beneficial effects of an intensified exercise program. Step length has been chosen as an outcome in previous studies examining the effects of LSVT-BIG.^43^ After the intervention improvements of 7.1 cm were reported, which is similar to improvements in our study in the BIG (+6.8 cm) and INTENSIVE groups (+6.6 cm). For the chair rising test, the cut-off value for a higher risk of falls is set at 16 seconds.^44^ A clinically meaningful change is detected by an improvement of 2.5 seconds.^45^ While the results of the LSVT-BIG group (−2.74 s) exceeded this value, improvements in the INTENSIVE group (−2.19 s) are similar and might also be considered meaningful. It has to be noted that none of the groups had a higher risk of falls at baseline. Taken together, these results yield improved general motor affection (UPDRS III) as well as the risk of falls (chair rising test) with a significant impact on gait parameters (step lengths) in the BIG and INTENSIVE groups. Significant changes in quality of life (PDQ-39) were not observed but numerical improvements in the PDQ-39 score in all groups exceeded the suggested clinically relevant change in PDQ-39 of −1.6 points over a 6-month time by Peto and colleagues.^46^ However, it has to be noted that the secondary outcome results are explorative as they were not corrected for multiple testing.

The effects of LSVT BIG therapy on motor functioning have been shown to be superior in comparison to Nordic walking or domestic home training.^47^ The current study aimed to compare LSVT BIG to an intensified physiotherapeutic training program that was delivered equally in terms of 16 individual 1-hour sessions over 4 weeks. Both groups ameliorated primary as well as secondary outcome parameters. No statistically significant differences between the two groups were found. These results are in accordance with a previous prospective, double-blinded and randomized clinical trial on LSVT BIG and general exercise in 11 patients with PD, reporting improved motor and non-motor scores in both groups after 6 months without significant group differences.^48^ The findings suggest an intensified physiotherapeutic training program to be equally effective for PD patients not able to access outpatient LSVT BIG therapy. This corroborates the hypothesis that improvements in motor symptoms by LSVT BIG are reached through higher attention within the time-consuming training setting and not mainly because of special LSVT BIG exercises.

With most studies suggesting that active intervention programs promote improvements in both motor and NMSs there is still a lack of randomized, controlled studies offering information about the most effective form of exercise and the right intervention frequency and period. Current clinical trials conducted two up to five sessions per week each lasting between 20 and 90 minutes. The reported duration periods varied greatly between 2 weeks (short LSVT BIG training protocol) and 3 years (active theatre training) with most frequent intervention periods lasting 4 up to 12 weeks.^35,43^ The outcomes of the present study indicate that an intensive, individualized 4-week physiotherapeutic training program might be a more important factor with regard to short-term improvements of motor and NMSs than an 8-week standard physiotherapy setting with the same amount of individual therapist time. Compared to LSVT BIG, intensive physiotherapy was not significantly more effective in reducing NMSs (despite higher within-group changes). Interestingly, in our study between-group differences of LSVT BIG and NORMAL after 8 weeks were highly non-significant (*p* = 1.000). Therefore, these results do not support the importance of high intensity training in general, but specifically emphasize the benefits of highly intensive, individualized physiotherapy training.

Future studies have to address how far these short-term effects can be observed also months after therapy. While physical therapy can improve long-term motor symptoms and physical functioning in PD, it remains unclear if or why more comprehensive training programs are beneficial with regard to long-term improvements of non-motor symptoms. However, possible advantages of high-intensity training might be cost effectiveness, increased patient motivation to practice independently at home (caused by a high perceived benefit) or training-induced neuroplasticity.^49^ More highly qualitative research is needed to evaluate the mechanisms of physiotherapeutic long-term effects in PD. Furthermore, it has to be noted that differences in outcome parameters of the present study may be influenced by varying therapeutic settings, in this study private practice *versus* hospital-based physiotherapeutic training.

Due to a limited study period, we missed the calculated sample size of 18 participants per group. This limits the generalizability of the results and reduces the power of the study. Furthermore, the common factor model in psychotherapy conjectures that relationship factors such as therapeutic alliance, positive regard, empathy and goal consensus between therapist and patient are important to the success of treatments and predicting outcomes.^50^ Therefore, non-specific parameters such as more skilled therapists, higher motivation, more intense attention and positive feedback in LSVT BIG and INTENSIVE as well as biased expectations of patients depending on participation in normal or intense training might have contributed to differences in change of outcome parameters between groups. Insufficient blinding might have contributed to these differences. Limited to the focus on short-term effects of these three exercise models, the study also addresses mainly global NMSs in PD. Future studies should aim also to investigate the effects of exercise modalities on more specific NMSs such as sleep disorders, mood disturbances, sensory deficits, autonomic dysfunction and cognitive deficits in more detail to tailor exercise better to patients’ individual symptoms. Finally, the possible participation in intensive training programs like LSVT BIG or INTENSIVE presumes a high level of motivation, general mobility and self-initiative. While missing records of training adherence rates for the groups of LSVT BIG and INTENSIVE after week 4 also have to be considered, this might have led to a selection bias, as individuals who opted to participate in the study might have had a more active lifestyle and were less impacted by motor and non-motor dysfunctions than the average PD patients in Hoehn & Yahr stages I to III also potentially limiting the generalizability of the findings.

## Supplemental Material

sj-pdf-1-tan-10.1177_1756286420986744 – Supplemental material for Effects of Lee Silverman Voice Treatment BIG and conventional physiotherapy on non-motor and motor symptoms in Parkinson’s disease: a randomized controlled study comparing three exercise models Click here for additional data file. Supplemental material, sj-pdf-1-tan-10.1177_1756286420986744 for Effects of Lee Silverman Voice Treatment BIG and conventional physiotherapy on non-motor and motor symptoms in Parkinson’s disease: a randomized controlled study comparing three exercise models by Fabian Schaible, Franziska Maier, Timo Marcel Buchwitz, Frank Schwartz, Marius Hoock, Eckhard Schönau, Miriam Libuda, Anke Hordt, Thilo van Eimeren, Lars Timmermann and Carsten Eggers in Therapeutic Advances in Neurological Disorders

sj-pdf-2-tan-10.1177_1756286420986744 – Supplemental material for Effects of Lee Silverman Voice Treatment BIG and conventional physiotherapy on non-motor and motor symptoms in Parkinson’s disease: a randomized controlled study comparing three exercise models Click here for additional data file. Supplemental material, sj-pdf-2-tan-10.1177_1756286420986744 for Effects of Lee Silverman Voice Treatment BIG and conventional physiotherapy on non-motor and motor symptoms in Parkinson’s disease: a randomized controlled study comparing three exercise models by Fabian Schaible, Franziska Maier, Timo Marcel Buchwitz, Frank Schwartz, Marius Hoock, Eckhard Schönau, Miriam Libuda, Anke Hordt, Thilo van Eimeren, Lars Timmermann and Carsten Eggers in Therapeutic Advances in Neurological Disorders
