# Supplementary material for: Effects of Lee Silverman Voice Treatment BIG and conventional physiotherapy on non-motor and motor symptoms in Parkinson’s disease: a randomized controlled study comparing three exercise models
Source: Ther Adv Neurol Disord. 2021 Feb 18;14:1756286420986744. doi: 10.1177/1756286420986744 (PMC7897809; doi:10.1177/1756286420986744)
Supplement: sj-pdf-1-tan-10.1177_1756286420986744 – Supplemental material for Effects of Lee Silverman Voice Treatment BIG and conventional physiotherapy on non-motor and motor symptoms in Parkinson’s disease: a randomized controlled study comparing three exercise models [file sj-pdf-1-tan-10.1177_1756286420986744.pdf]

SAGE AUTHORSHIP CHANGE REQUEST

INSTRUCTIONS:
Please print and complete all sections of this form. We require that all authors (including current co-authors, those to be added and those to be removed) sign the relevant sections below. Once complete, please scan the form and return to <<email address>>

SECTION ONE: PAPER INFORMATION

JOURNAL NAME	Therapeutic Advances in Neurological Disorders
PAPER TITLE	MANUSCRIPT ID NUMBER
Effects of LSVT BIG and conventional physiotherapy on non-motor and motor symptoms in Parkinson's disease: a randomized controlled study comparing three exercise models	TAN-20-OR-0015.R1

SECTION TWO: ESSENTIAL CONDITIONS FOR CHANGE
The new author list should contain only those who can legitimately claim authorship. This is all those who:

1) Have made a substantial contribution to the concept and design, acquisition of data or analysis and interpretation of data; AND
2) Drafted the article or revised it critically for important intellectual content; AND
3) Approved the version to be published.

NB: Registered authors should meet the conditions of all of the points above. All contributors who do not meet the criteria for authorship should instead be listed in an `Acknowledgements' section.

SECTION THREE: REASON FOR AUTHORSHIP CHANGE

Please provide a detailed explanation for the change in authors in the box below.

SAGE

To incorporate the aspects mentioned by the reviewers into the manuscript, additional statistical analyses as well as revision of theoretical content was necessary. As expected this process required more than a few hours of work. Due to time constraints caused by other projects and the holiday season none of the previously enlisted authors were able to meet these requirements. Therefore, Timo Marcel Buchwitz joined the research team and took care of most of the review process. Because of his substantial contribution to the manuscript we would like to add him to the list of authors.

SAGE

SECTION FOUR: AUTHOR DECLARATIONS

We agree to the proposed change of authorship to the above paper. NB: all authors (added and removed) must sign below

DECLARATION: All the below listed authors agree that they can claim authorship to the submitted paper as they meet the criteria of the points listed in SECTION TWO.

List of existing and proposed new authors to sign. Please sign, indicating your agreement to the proposed change. The individuals listed below will each be credited as authors in the published paper. (Please add additional rows if required.)		
Author name	Author signature	Date
Fabian Schaible, MD		
Franziska Maier, MD		
Timo Marcel Buchwitz	[illegible]	08.07.20
Frank Schwartz, MD		
Marius Hoock, MD		
Eckhard Schönau, MD		
Miriam Libuda		
Anke Hordt		
Thilo van Eimeren, MD		
Lars Timmermann, MD	[illegible]	08/07/20
Carsten Eggers, MD	[illegible]	08.07.20

SAGE

SECTION FOUR: AUTHOR DECLARATIONS

We agree to the proposed change of authorship to the above paper. NB: all authors (added and removed) must sign below

DECLARATION: All the below listed authors agree that they can claim authorship to the submitted paper as they meet the criteria of the points listed in SECTION TWO.

List of existing and proposed new authors to sign. Please sign, indicating your agreement to the proposed change. The individuals listed below will each be credited as authors in the published paper. (Please add additional rows if required.)		
Author name	Author signature	Date
Fabian Schaible, MD		
Franziska Maier, MD		
Timo Marcel Buchwitz	[illegible]	05.07.20
Frank Schwartz, MD		
Marius Hoock, MD		
Eckhard Schönau, MD		
Miriam Libuda	[illegible]	10.7.20
Anke Hordt	A. Hordt	13.7.20
Thilo van Eimeren, MD		
Lars Timmermann, MD		
Carsten Eggers, MD	[illegible]	05.07.20

SAGE

SECTION FOUR: AUTHOR DECLARATIONS

We agree to the proposed change of authorship to the above paper. NB: all authors (added and removed) must sign below

DECLARATION: All the below listed authors agree that they can claim authorship to the submitted paper as they meet the criteria of the points listed in SECTION TWO.

List of existing and proposed new authors to sign. Please sign, indicating your agreement to the proposed change. The individuals listed below will each be credited as authors in the published paper. (Please add additional rows if required.)		
Author name	Author signature	Date
Fabian Schaible, MD	[illegible]	11.07.20
Franziska Maier, MD		
Timo Marcel Buchwitz	[illegible]	08.07.20
Frank Schwartz, MD		
Marius Hoock, MD		
Eckhard Schönau, MD		
Miriam Libuda		
Anke Hordt		
Thilo van Eimeren, MD		
Lars Timmermann, MD		
Carsten Eggers, MD	[illegible]	08.07.20

SECTION FOUR: AUTHOR DECLARATIONS

We agree to the proposed change of authorship to the above paper. NB: all authors (added and removed) must sign below

DECLARATION: All the below listed authors agree that they can claim authorship to the submitted paper as they meet the criteria of the points listed in SECTION TWO.

List of existing and proposed new authors to sign. Please sign, indicating your agreement to the proposed change. The individuals listed below will each be credited as authors in the published paper. (Please add additional rows if required.)		
Author name	Author signature	Date
Fabian Schaible, MD		
Franziska Maier, MD		
Timo Marcel Buchwitz	[illegible]	[illegible].07.20
Frank Schwartz, MD		
Marius Hoock, MD		
Eckhard Schönau, MD		
Miriam Libuda		
Anke Hordt		
Thilo van Eimeren, MD	[illegible]	08/07/20
Lars Timmermann, MD	[illegible]	08/07/20
Carsten Eggers, MD	[illegible]	08.07.20

SAGE

SECTION FOUR: AUTHOR DECLARATIONS

We agree to the proposed change of authorship to the above paper. NB: all authors (added and removed) must sign below

DECLARATION: All the below listed authors agree that they can claim authorship to the submitted paper as they meet the criteria of the points listed in SECTION TWO.

List of existing and proposed new authors to sign. Please sign, indicating your agreement to the proposed change. The individuals listed below will each be credited as authors in the published paper. (Please add additional rows if required.)		
Author name	Author signature	Date
Fabian Schaible, MD		
Franziska Maier, MD		
Timo Marcel Buchwitz	T. Buchwitz	08.07.20
Frank Schwartz, MD		
Marius Hoock, MD	M. Hoock	14.07.20
Eckhard Schönau, MD		
Miriam Libuda		
Anke Hordt		
Thilo van Eimeren, MD		
Lars Timmermann, MD		
Carsten Eggers, MD	C. Eggers	05.07.20

SECTION FOUR: AUTHOR DECLARATIONS

We agree to the proposed change of authorship to the above paper. NB: all authors (added and removed) must sign below

DECLARATION: All the below listed authors agree that they can claim authorship to the submitted paper as they meet the criteria of the points listed in SECTION TWO.

List of existing and proposed new authors to sign. Please sign, indicating your agreement to the proposed change. The individuals listed below will each be credited as authors in the published paper. (Please add additional rows if required.)

Author name	Author signature	Date
Fabian Schaible, MD		
Franziska Maier, MD		
Timo Marcel Buchwitz		05.07.20
Frank Schwartz, MD		
Marius Hoock, MD		
Eckhard Schönau, MD		13.7.20
Miriam Libuda		
Anke Hordt		
Thilo van Eimeren, MD		
Lars Timmermann, MD		
Carsten Eggers, MD		05.07.20

SAGE

SECTION FOUR: AUTHOR DECLARATIONS

We agree to the proposed change of authorship to the above paper. NB: all authors (added and removed) must sign below

DECLARATION: All the below listed authors agree that they can claim authorship to the submitted paper as they meet the criteria of the points listed in SECTION TWO.

List of existing and proposed new authors to sign. Please sign, indicating your agreement to the proposed change. The individuals listed below will each be credited as authors in the published paper. (Please add additional rows if required.)		
Author name	Author signature	Date
Fabian Schaible, MD		
Franziska Maier, MD		13.07.2020
Timo Marcel Buchwitz		08.07.20
Frank Schwartz, MD		
Marius Hoock, MD		
Eckhard Schönau, MD		
Miriam Libuda		
Anke Hordt		
Thilo van Eimeren, MD		
Lars Timmermann, MD		
Carsten Eggers, MD		08.07.20

SECTION FOUR: AUTHOR DECLARATIONS

We agree to the proposed change of authorship to the above paper. NB: all authors (added and removed) must sign below

DECLARATION: All the below listed authors agree that they can claim authorship to the submitted paper as they meet the criteria of the points listed in SECTION TWO.

List of existing and proposed new authors to sign. Please sign, indicating your agreement to the proposed change. The individuals listed below will each be credited as authors in the published paper. (Please add additional rows if required.)		
Author name	Author signature	Date
Fabian Schaible, MD		
Franziska Maier, MD		
Timo Marcel Buchwitz		[illegible]
Frank Schwartz, MD		11.07.2020
Marius Hoock, MD		
Eckhard Schönau, MD		
Miriam Libuda		
Anke Hordt		
Thilo van Eimeren, MD		
Lars Timmermann, MD		
Carsten Eggers, MD		15.07.20

DECLARATION: All the below listed authors agree that they cannot claim authorship to the submitted paper as they do not (or no longer) meet the criteria of the points listed in SECTION TWO.

List of removed authors. Please sign, indicating your agreement to be removed from the list of registered authors. The individuals listed below will not be credited as authors in the published paper. (Please add additional rows if required.)		
Author name	Author signature	Date
