# Supplementary material for: Effects of Lee Silverman Voice Treatment BIG and conventional physiotherapy on non-motor and motor symptoms in Parkinson’s disease: a randomized controlled study comparing three exercise models
Source: Ther Adv Neurol Disord. 2021 Feb 18;14:1756286420986744. doi: 10.1177/1756286420986744 (PMC7897809; doi:10.1177/1756286420986744)

Exercise for Mobility Nr. 1

Starting Position:

- Supine position, legs raised.

Execution:

- Put your legs down alternately to the left and right side.

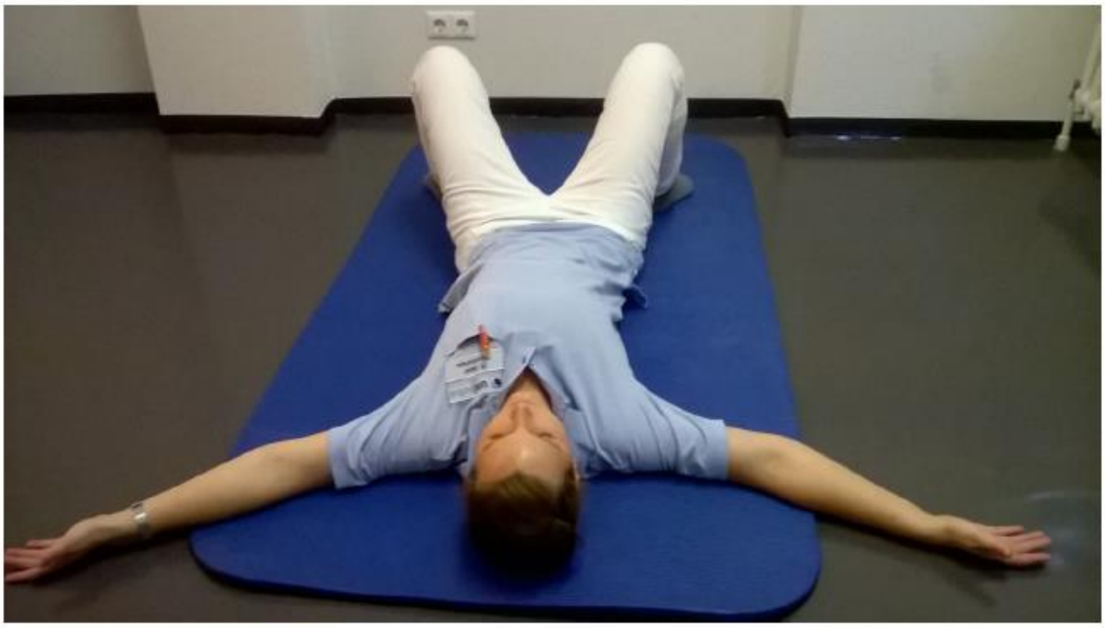

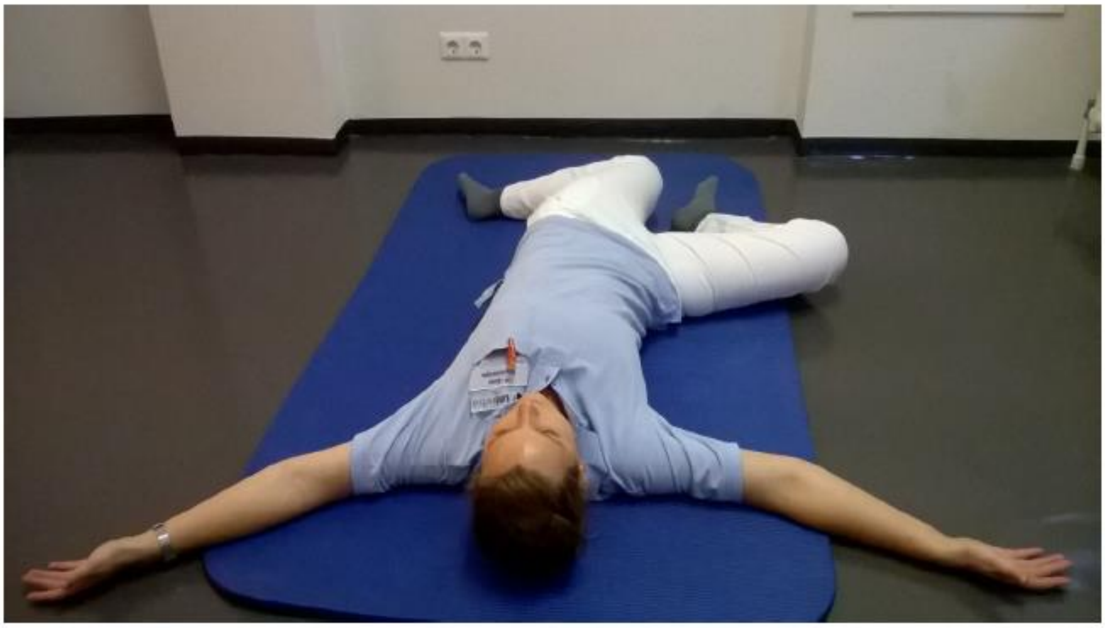

Exercise for Mobility Nr. 2

Starting Position:

- Lateral Position, legs are bent and lie on top of each other.

Execution:

- Put your stretched arms in front of your shoulder.
- Stretch your upper hand forward as much as possible.
- Raise the arm through the air, over your shoulder, until you can put it on the ground.
- Your head and upper body are rotating. If possible, your legs remain lying.
- Repeat this exercise 10 times. Then rotate to the other side and repeat the exercise with your other arm.

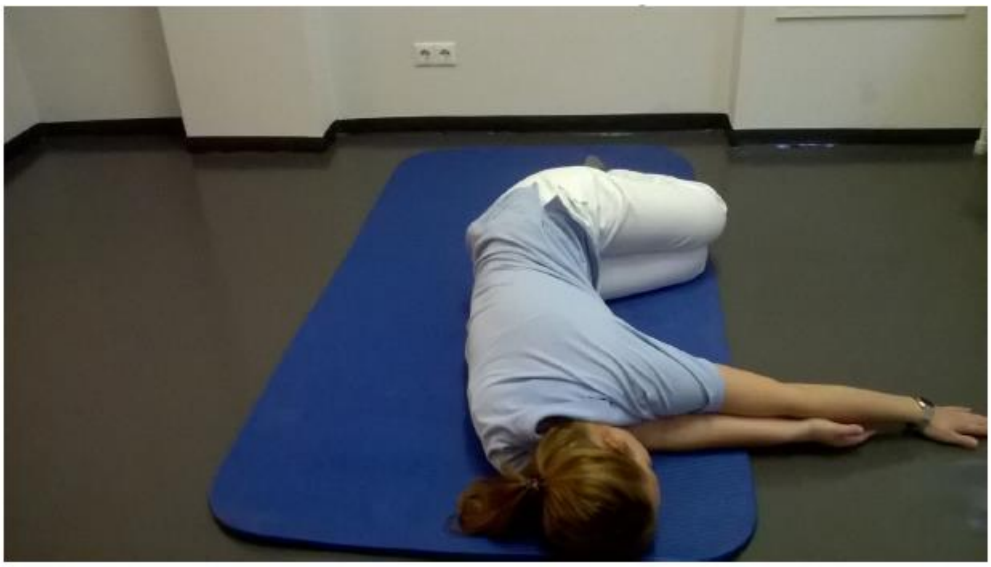

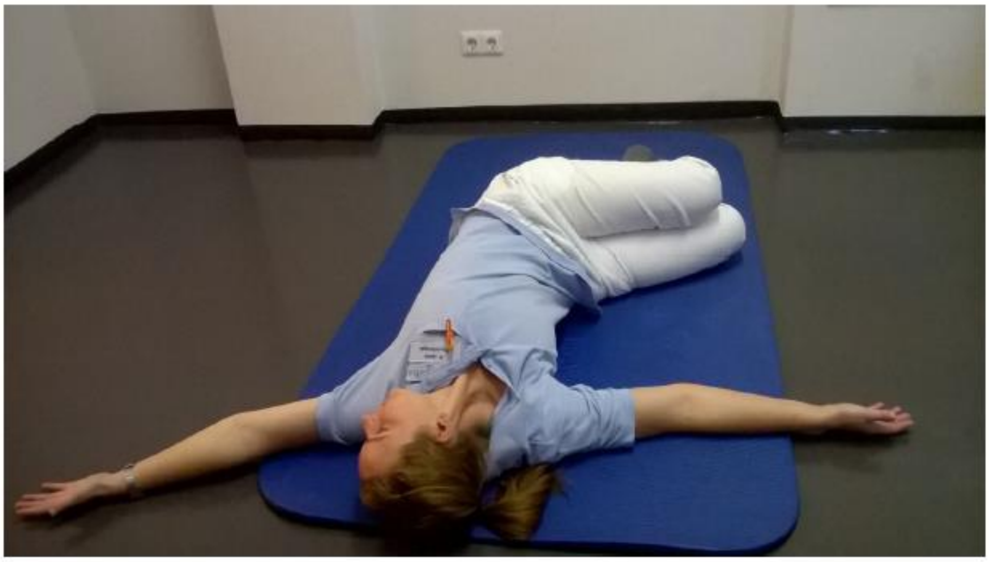

Exercise for Mobility Nr. 3

Starting Position:

- Take a chair and place it in front of a wall with the seat pointing forward (to prevent it from slipping away).
- Hold on to the chair back.

Execution:

- Walk backwards until your hips are in a right angle (90°).
- Straighten your back. Stretch your shoulders and the back side of your legs by doing so.
- Repeat this exercise 10 times.

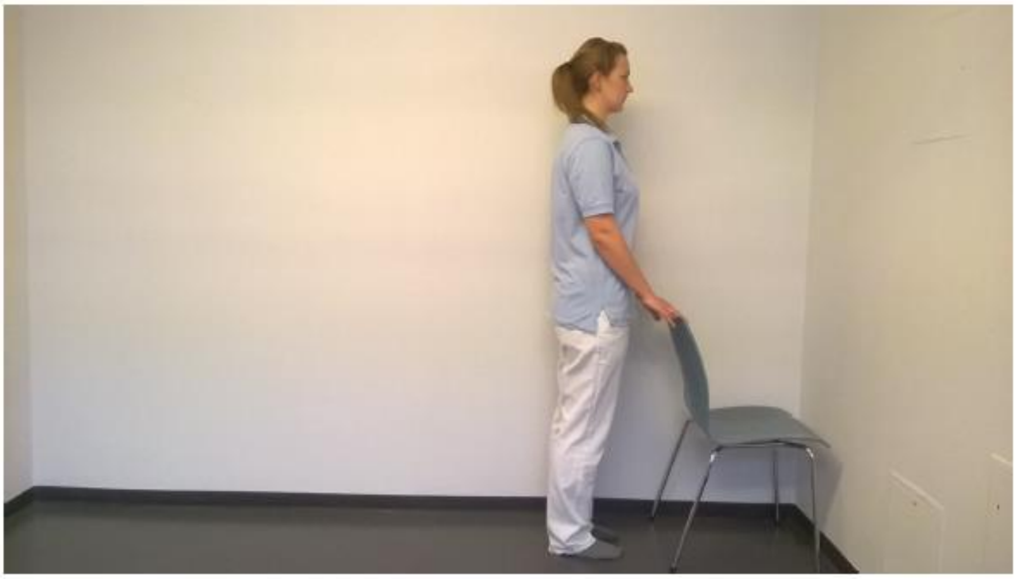

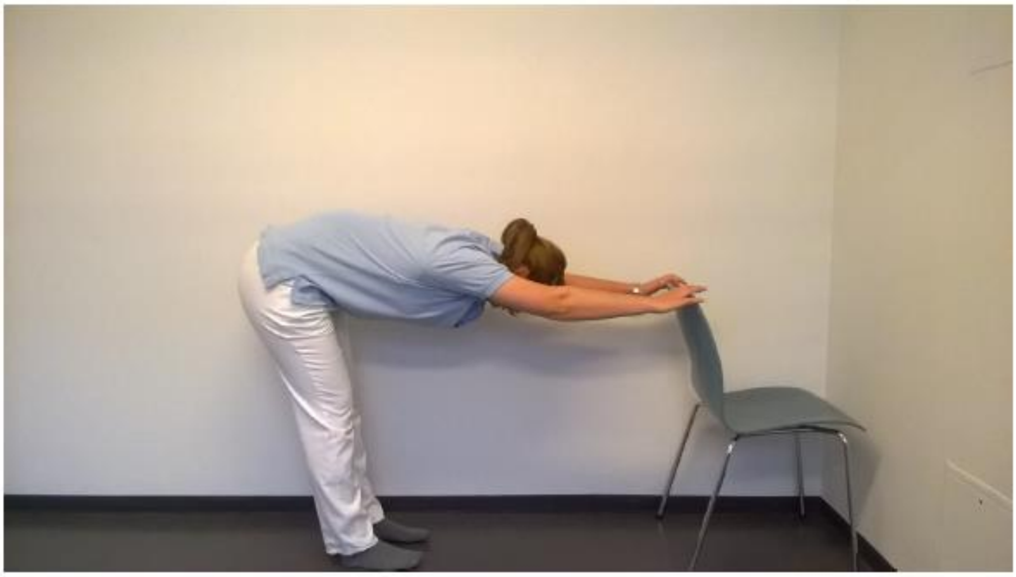

Exercise for Mobility Nr. 4

Starting Position:

- Raise one hand into the air to hold on to a doorframe.

Execution:

- Cling to your hand by moving your pelvis further to the side. You may feel a stretch in your body side.
- Repeat this exercise 10 times. Then change sides.

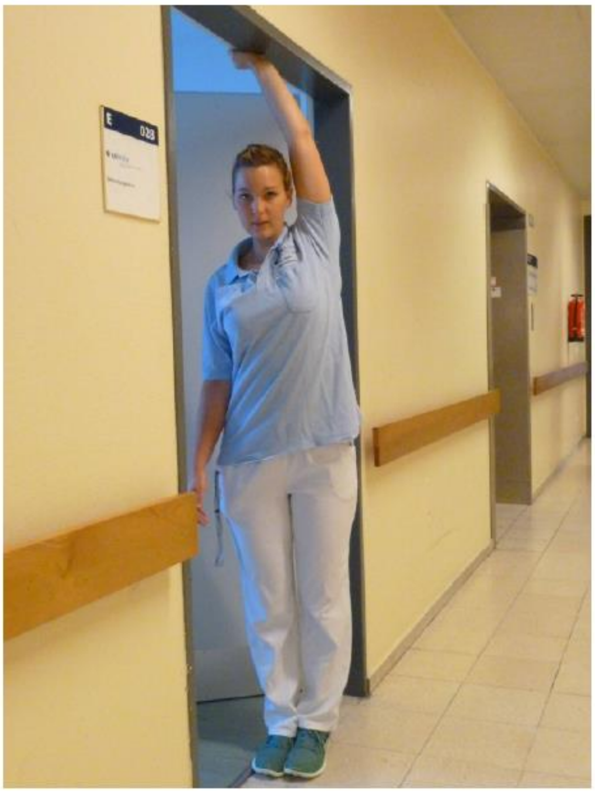

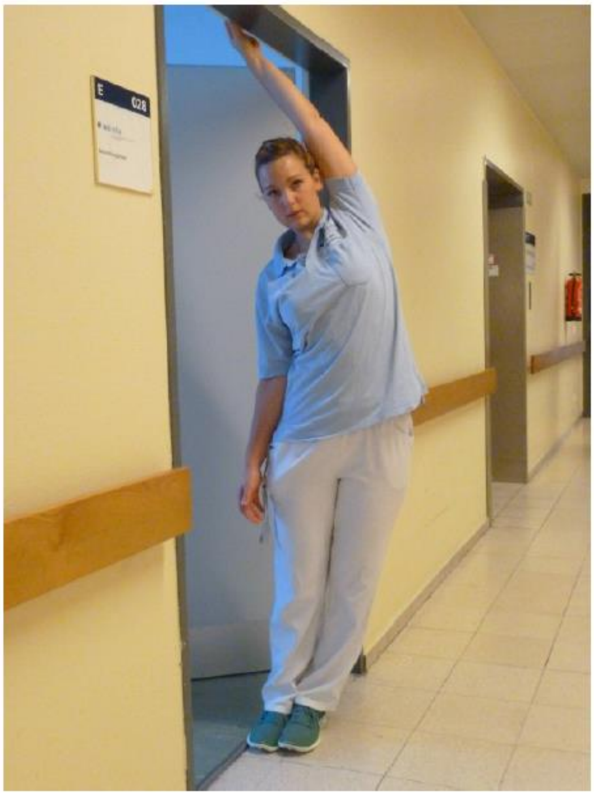

Exercise for Mobility Nr. 5

Starting Position:

- Quadruped stand.

Execution:

- Stretch your right foot backwards as far as possible.
- By doing so also stretch your hip and breast muscles.
- Then pull your right knee towards your left elbow (as far as possible) to stretch your back.
- Repeat this exercise 5 times on each side.

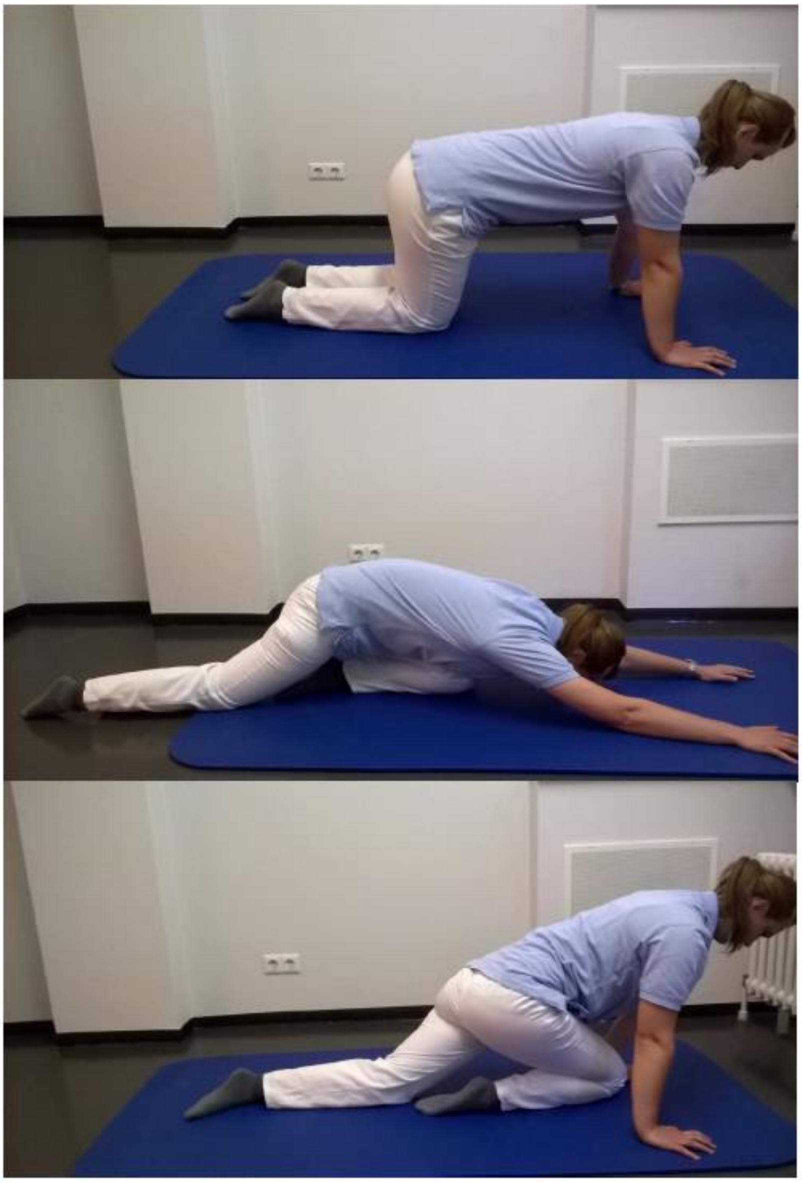

Exercise for Mobility Nr. 6

Starting Position:

- Stomach position. Straighten your arms and put them next to your shoulders.

Execution:

- Lift your straightened left leg from the ground until your thigh is in the air.
- Try to move your left leg to the right side to stretch your front left side and your hip flexor muscles.
- Change sides and repeat the exercise.
- Perform the exercise 5 times on each side.

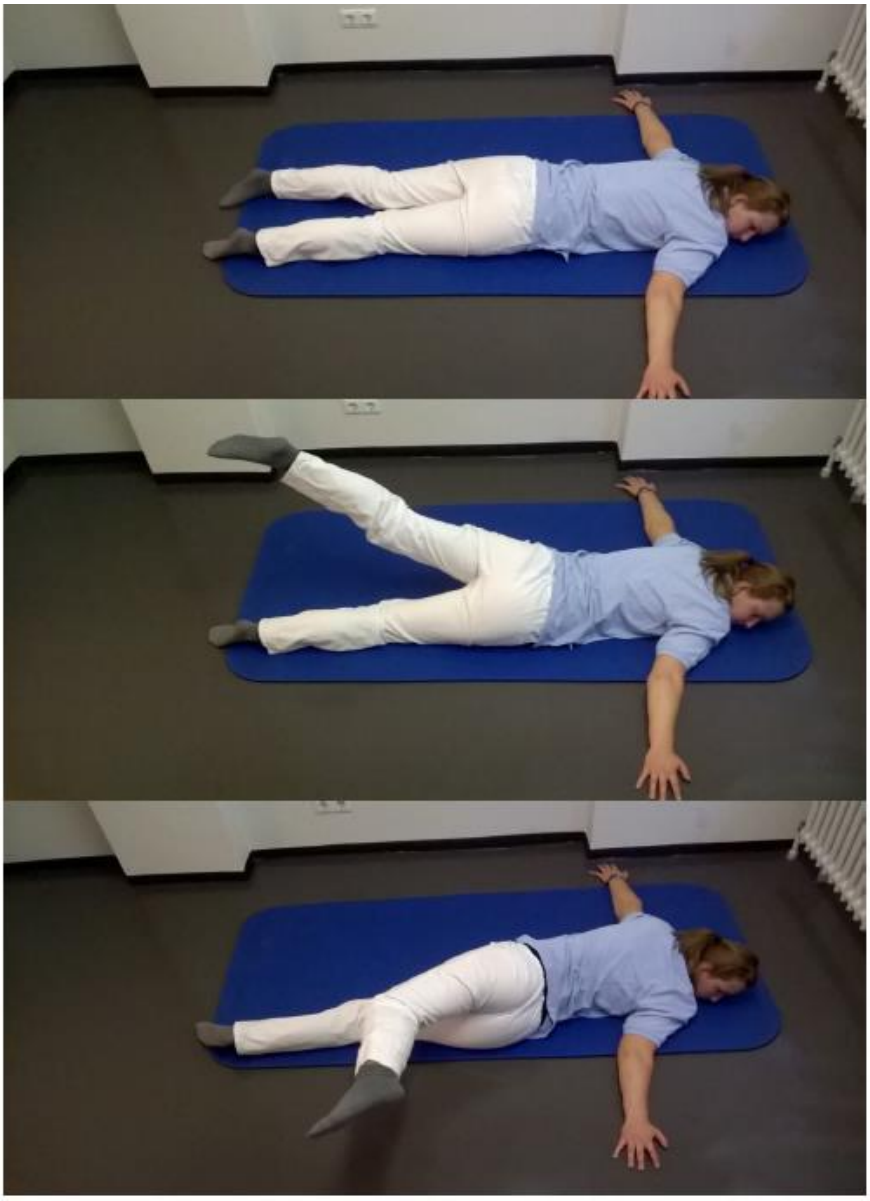

Supplement: sj-pdf-2-tan-10.1177_1756286420986744 – Supplemental material for Effects of Lee Silverman Voice Treatment BIG and conventional physiotherapy on non-motor and motor symptoms in Parkinson’s disease: a randomized controlled study comparing three exercise models [file sj-pdf-2-tan-10.1177_1756286420986744.pdf]
